# Genetic identification of SNP markers linked to a new grape phylloxera resistant locus in *Vitis cinerea* for marker-assisted selection

**DOI:** 10.1186/s12870-018-1590-0

**Published:** 2018-12-18

**Authors:** Harley M. Smith, Catherine W. Clarke, Brady P. Smith, Bernadette M. Carmody, Mark R. Thomas, Peter R. Clingeleffer, Kevin S. Powell

**Affiliations:** 1 CSIRO Agriculture and Food, Glen Osmond, SA 5064 Australia; 2 Agriculture Victoria, Biosciences Research Division, 124 Chiltern Valley Road, Rutherglen, Melbourne, Victoria 3685 Australia; 3 Sugar Research Australia, PO Box 122, Gordonvale, QLD 4865 Australia; 4 The Australian Wine Research Institute, Box 197, Glen Osmond, SA 5064 Australia

**Keywords:** Phylloxera, Grapevine, Rootstock, Single nucleotide polymorphism (SNP), Marker-assisted breeding

## Abstract

**Background:**

Grape phylloxera (*Daktulosphaira vitifoliae* Fitch) is a major insect pest that negatively impacts commercial grapevine performance worldwide. Consequently, the use of phylloxera resistant rootstocks is an essential component of vineyard management. However, the majority of commercially available rootstocks used in viticulture production provide limited levels of grape phylloxera resistance, in part due to the adaptation of phylloxera biotypes to different *Vitis* species. Therefore, there is pressing need to develop new rootstocks better adapted to specific grape growing regions with complete resistance to grape phylloxera biotypes.

**Results:**

Grapevine rootstock breeding material, including an accession of *Vitis cinerea* and *V. aestivalis*, DRX55 ([*M. rotundifolia* x *V. vinifera*] x open pollinated) and MS27-31 (*M. rotundifolia* specific hybrid), provided complete resistance to grape phylloxera in potted plant assays. To map the genetic factor(s) of grape phylloxera resistance, a F_1_ *V. cinerea* x *V. vinifera* Riesling population was screened for resistance. Heritability analysis indicates that the *V. cinerea* accession contained a single allele referred as *RESISTANCE TO DAKTULOSPHAIRA VITIFOLIAE 2* (*RDV2*) that confers grape phylloxera resistance. Using genetic maps constructed with pseudo-testcross markers for *V. cinerea* and Riesling, a single phylloxera resistance locus was identified in *V. cinerea*. After validating SNPs at the *RDV2* locus, interval and linkage mapping showed that grape phylloxera resistance mapped to linkage group 14 at position 16.7 cM.

**Conclusion:**

The mapping of *RDV2* and the validation of markers linked to grape phylloxera resistance provides the basis to breed new rootstocks via marker-assisted selection that improve vineyard performance.

**Electronic supplementary material:**

The online version of this article (10.1186/s12870-018-1590-0) contains supplementary material, which is available to authorized users.

## Background

Grape phylloxera (*Daktulosphaira vitifoliae* Fitch) is an insect native to specific regions of North America and *Vitis vinifera* cultivars used in grape production are highly susceptible to this insect pest [1–3]. In the mid-1800s, the accidental introduction of grape phylloxera from North America to Europe nearly destroyed the European wine industry [2, 4]. However, the identification of North American *Vitis* species that evolved resistance to grape phylloxera were utilized as rootstocks to re-establish wine grape production in Europe. In addition to Europe, grape phylloxera spread to other wine grape growing regions of the world including South Africa, Middle East, Asia and Australasia in the mid to late 1800s [4]. Interestingly, while the life cycle of grape phylloxera was originally classified as cyclic parthenogenesis, which alternates between sexual and asexual forms [1], studies in North America, Europe and Australia indicate that phylloxera reproduction predominantly occurs asexually [2, 5].

Grape phylloxera genetic strains that feed primarily on *Vitis* spp. roots or leaves are referred to as radicicoles or gallicoles, respectively. In some cases, the radicicole and gallicole strains will feed on both roots and leaves. Once feeding is established, cells surrounding the feeding site undergo cell proliferation and expansion to form a gall [1, 3]. Histological and gene expression studies performed on root and leaf galls indicate that these feeding structures act as nutrient sinks necessary for phylloxera growth, development and reproduction [6–9]. In terms of viticulture production, radicicoles are the most destructive form of grape phylloxera due to root damage caused by gall formation and feeding, as well as the fact that gallicole feeding is relatively rare in *V. vinifera* [1, 3].

Three types of root galls develop in response to grape phylloxera feeding and the type of swelling produced is based on the genotype of the insect and host plant. The most common gall that develops on all susceptible *Vitis* species and hybrids are called nodosities, which are characterized as hooked galls that form near the tip of actively growing non-lignified immature roots [1, 3]. The degree of nodosity formation can often vary across *Vitis* species and hybrids, such that rootstocks with a low level of nodosity formation are often characterized as resistant or tolerant. In addition to nodosity formation, grape phylloxera feeding on older lignified roots of *V. vinifera* cultivars and hybrids will give rise to root swellings called tuberosities [3, 10, 11]. As tuberosities develop, they are prone to cracking, which provides an entry point for soil borne fungal pathogens that severely damages the root system causing necrosis and can eventually result in vine death [12, 13]. Lastly, grape phylloxera genetic strains categorized as biotype C not only induce nodosities on young roots, but also have the capacity to form non-necrotic swellings on mature roots, termed pseudotuberosities in *V. riparia* [14–16]. Pseudotuberosities are categorised as dome-shaped swellings on lignified roots which, unlike tuberosities, have no root cracking or subsequent necrotic regions caused by fungal entry and establishment [17]. The emergence of biotype C genetic strains adapted to feeding on *V. riparia* rootstocks is of concern when selecting for the appropriate rootstock in phylloxera-infested regions due to the potential for rootstock failure. In vineyards using own rooted *V. vinifera* cultivars, feeding damage induced by radicicoles reduces the roots’ ability to uptake water and nutrients resulting in yield losses [3]. Moreover, It has been estimated that vine death can occur within a 4-7 year period in vineyards infested with highly aggressive genetic strains [3].

Utilization of rootstocks having a high level of resistance to grape phylloxera biotypes is an essential component for maintaining production in the presence of this insect pest. Initial breeding efforts for grape phylloxera resistance produced rootstocks that supported a low level of phylloxera feeding and reproduction with minimal damage to the root system. For example, AxR#1, a *V. vinifera* Aramon x *V. rupestris* Ganzin hybrid was widely used in California, as the phylloxera genetic strain classified as ‘biotype A’ performed poorly on this rootstock [18]. In addition, ‘biotype A’ phylloxera also performed poorly on *V. berlandieri* x *V. riparia* rootstocks, including Teleki 5C, SO4, 5BB Kober and 420A, in California, Europe and Australia [3, 4, 19, 20]. However, widespread usage of AxR#1 in California and *V. berlandieri* x *V. riparia* rootstocks in Europe resulted in the emergence of grape phylloxera biotypes B and C, respectively, which are adapted to feeding and reproducing on these rootstocks [18–20]. In California, failure of the AxR#1 rootstock due to the emergence of the biotype B grape phylloxera cost the wine industry up to $1.25 billion [21]. Therefore, selection pressure resulting from wide spread usage of partially resistant rootstocks with a minimum spectrum of protection can lead to the emergence of less abounding grape phylloxera biotypes and the eventual breakdown of resistance.

Heritability studies have identified sources of grape phylloxera resistance for rootstock breeding; however, the number of loci controlling the resistance appears to vary. For example, evaluation of grape phylloxera resistance in F_1_ hybrids from *V. vinifera* x *M. rotundifola* showed that resistance segregates with a 1:1 ratio indicating that a single locus confers phylloxera resistance in this *M. rotundifolia* accession [22]. However, when two resistant F_1_ individuals were backcrossed to *V. vinifera*, only 19% of the individuals had complete resistance. As a result, Bouquet, 1983, concluded that grape phylloxera resistance in the *M. rotundifolia* is mediated by a semi-dominant locus, which is regulated by three genetic modifiers [22]. In another study, analysis of grape phylloxera resistance in F_1_ *V. vinifera* x *M. rotundifolia* individuals indicated that resistance is controlled by more than one locus in an *M. rotundifolia* accession [23]. Using a design II mating scheme, grape phylloxera resistance was examined in seven rootstocks with different levels of resistance and susceptibility and results indicated that resistance is controlled by at least two loci [24]. In *V. berlandieri* and *V. cinerea*, resistance appears to be controlled by multiple loci [10, 25]. Genetic mapping studies showed that a major phylloxera resistant QTL, *RESISTANCE DAKTULOSPHAIRA VITIFOLIAE 1* (*RDV1*), is located on chromosome 13 in the Börner (*V. riparia* x *V. cinerea*) rootstock [25]. The *RDV1* locus appears to have originated from *V. cinerea*, as markers linked to this resistant locus were derived from this North American *Vitis* species. In addition, a single QTL that provides grape phylloxera resistance in leaves was identified and maps to chromosome 14 in the *Vitis* hybrid, MN1264, which has a pedigree containing *V. riparia*, *V. vinifera*, *V. labrusca*, *V. rupestris*, *V. aestivalis*, and *V. lincecumii* [26]. In addition, two loci that map to chromosome 5 and 10 in MN1264 and MN1246, respectively, mediate grape phylloxera resistance in roots. Due to the high heterozygosity of MN1264 and MN1246, it has yet to be determined which species contributed the leaf and root specific resistant loci [26].

In addition to grape phylloxera resistant alleles, heritability studies indicated that *V. vinifera* contain grape phylloxera susceptibility loci, which function to promote radicicole formation [10, 11]. Initial studies indicated that radicicole formation in *V. vinifera* was due to a single allele [10]. However, a later study examined this further by determining inheritance of nodosity and tuberosity formation [11]. Results from this study indicated that at least two loci control nodosity development in *V. vinifera*. However, at this time, the heritability of tuberosity formation is not clear [11].

To identify additional sources of grape phylloxera resistance for marker-assisted selection of new rootstocks, four grapevine species and hybrids were identified, including *V. cinerea* C2-50, which has complete resistance to two highly aggressive phylloxera strains. Heritability analysis indicates that a single locus in *V. cinerea* C2-50 confers resistance to two related grape phylloxera strains. Using linkage and interval mapping, a new phylloxera resistance locus was identified in *V. cinerea* C2-50. Validation of SNPs identified the position of the resistance locus on linkage group 14. Results from this study and Zhang et al., 2009 indicate that *V. cinerea* contains at least two phylloxera resistant loci and together the markers linked to these loci can be used in a marker assisted scheme to breed new rootstocks aimed at improving vineyard performance in the presence of this insect pest.

## Results

### Identification of rootstock breeding material with resistance to G1 grape phylloxera

Experimental evidence indicates that *V. cinerea*, *V. aestivalis* and *M. rotundifolia* accessions are sources of grape phylloxera resistance [10, 25, 27]. Based on these studies, *V. cinerea* C2-50 and an accession of *V. aestivalis*, as well as DRX55 ([*M. rotundifolia* x *V. vinifera*] x open pollinated), MS27-31 (*M. rotundifolia* interspecific hybrid) and 171-13L (*V. aestivalis* x *V. vinifera*) were screened for resistance to G1 grape phylloxera. As a control, Riesling and Shiraz were included in this screen, as these *V. vinifera* cultivars are highly susceptible to G1 grape phylloxera. Results showed that the *V. cinerea* C2-50 and *V. aestivalis* accessions, as well as DRX55 and MS27-31, were resistant to the G1 grape phylloxera genotype, as nodosity formation and insect development did not occur on the roots of these vines (Table 1). However, nodosity formation and insect development were detected on the roots of 171-13L, Riesling and Shiraz (Table 1). Due to difficulties with propagating progeny from *V. aestivalis* x *V. vinifera* and breeding with *M. rotundifolia* x *Vitis* spp. hybrids, we sought to determine the genetic determinants of phylloxera resistance in *V. cinerea* C2-50.

**Table 1:** Evaluation of rootstock breeding material for G1 grape phylloxera resistance

Variety/Accession	Pedigree	Nodosities	Insects	Rating
*V. cinerea* C2-50	*V. cinerea accession*	0	0	R
DRX55	(*V. vinifera* x *M. rotundifoli*a) open pollinated	0	0	R
MS27-31	*M. rotundifolia* interspecific hybrid	0	0	R
*V. aestivalis*	*V. aestivalis* accession	0	0	R
171-13L	*V. aestivalis* x *V. vinifera*	5.7	20.3	S
Riesling	*V. vinifera* variety	53.3	346.7	S
Shiraz	*V. vinifera* variety	96.7	596.7	S

The average number of nodosities and insects is displayed.

*R* resistant, *S* susceptible

### Segregation of grape phylloxera resistance in *V. cinerea* C2-50

The heritability of G1 grape phylloxera resistance in *V. cinerea* C2-50 was examined by screening 90 F_1_ *V. cinerea* C2-50 x Riesling individuals, which were recently genotyped [28]. Plants with nodosity formation and insect development on roots were classified as susceptible, as these two parameters indicate that G1 grape phylloxera can effectively feed and reproduce on these vines. In contrast, roots of vines devoid of nodosity formation and insect development were classified as resistant. Results showed that nodosity formation and insect development did not occur on the roots of 46 F_1_ *V. cinerea* C2-50 x Riesling individuals indicating that these progeny were resistant to G1 phylloxera (Table 2). In contrast, the remaining 44 F_1_ individuals had nodosity development and a significant number of insects were found on the roots (Table 2). The average number of nodosities and insects varied among the susceptible F_1_ individuals (Additional file 1). G4 grape phylloxera resistance was also examined by screening 58 out of the 90 F_1_ *V. cinerea* C2-50 x Riesling individuals for resistance and susceptibility. Results showed that 32 were resistant due to the absence of insects and nodosity development (Table 2). However, 26 individuals were susceptible, as nodosity formation and insect development were apparent (Table 2). As with the G1 phenotype, the average number of nodosities and G4 insects varied for the F_1_ individuals (Additional file 2). It should be pointed out that all 32 F_1_ individuals resistant to G4 grape phylloxera were also resistant to G1. In addition, the F_1_ plants susceptible to G4 grape phylloxera were effectively parasitized by G1. Therefore, the mode of G1 and G4 grape phylloxera resistance appears to be mediated by the same mechanism.

**Table 2:** Segregation of G1 and G4 grape phylloxera resistance

Grape Phylloxera Genetic Strain	R	S	Proposed R:S ratio	Calculated *X*^2^
G1	46	44	1:1	0.0444
G4	32	26	1:1	0.6207

The number of resistant (R) and susceptible (S) F_1_ individuals is displayed.

A chi-square goodness of fit test was performed to determine whether the phenotypic ratio for G1 and G4 grape phylloxera resistance segregates with a 1:1 ratio. Using one degree of freedom, the chi-square value for G1 and G4 was 0.0444 and 0.6207, respectively, and these values are less than the critical value of 3.84 (Table 2). In addition, the probability for each of the values was >0.05 indicating that G1 and G4 grape phylloxera resistance segregates with a 1:1 ratio. Therefore, the data support a model that G1 and G4 resistance, referred to as *RESISTANCE TO DAKTULOSPHAIRA VITIFOLIAE 2* (*RDV2)*, is conferred by a single allele in *V. cinerea* C2-50, which can be explained by two hypotheses. In the first hypothesis, *V. cinerea* C2-50 is heterozygous dominant for *RDV2* (*RDV2/rdv2*) and Riesling is homozygous recessive (*rdv2/rdv2*). Alternatively, the second hypothesis predicts that resistance is conferred by a recessive allele (*rdv2*) in which *V. cinerea* C2-50 is homozygous recessive (*rdv2/rdv2*) and Riesling is heterozygous (*RDV2/rdv2*) for the recessive allele.

### Genetic mapping of the *RDV2* locus

**Fig. 1:**
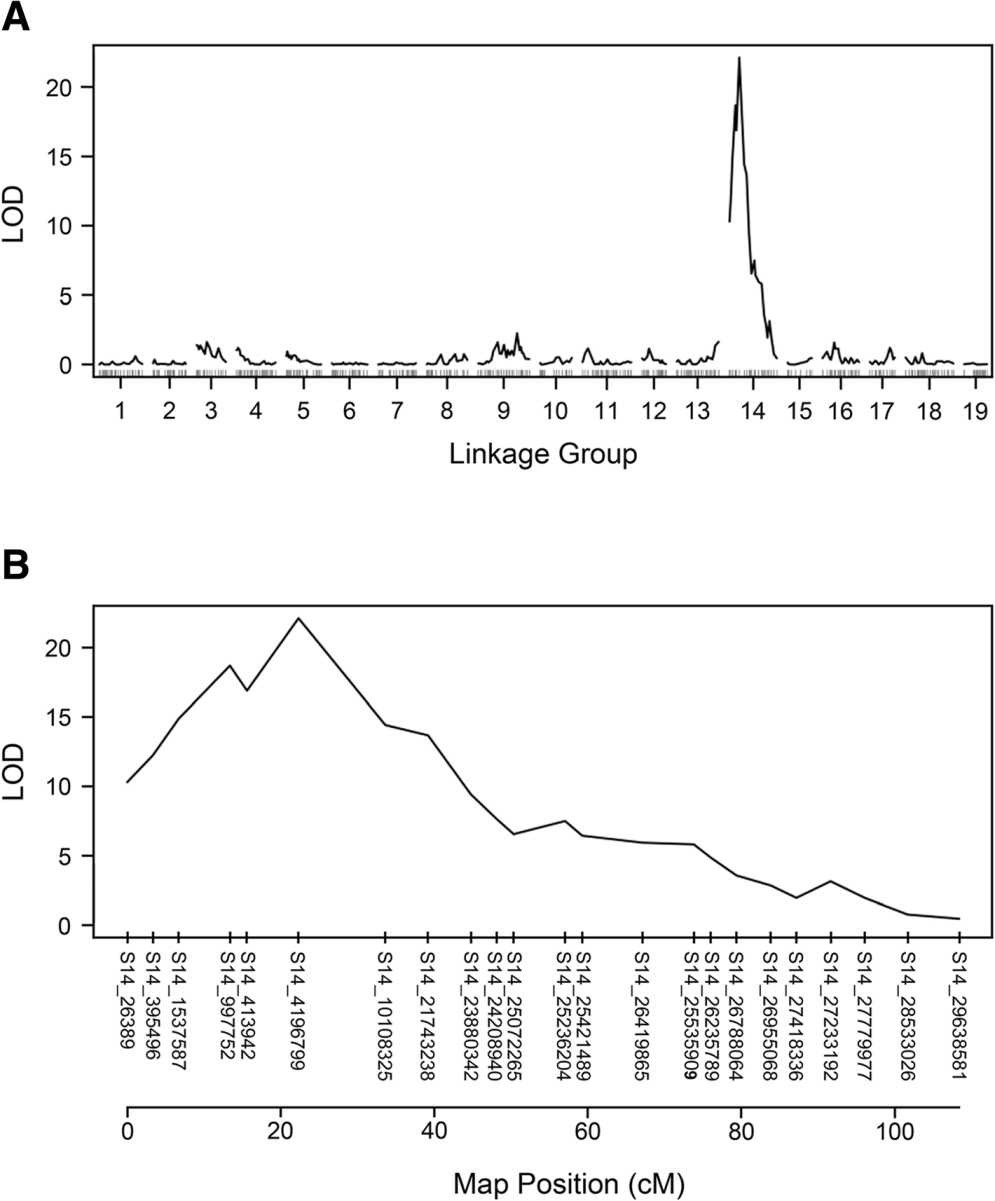
Interval mapping of grape phylloxera resistance using the C2-50 genetic map. (**a**) Using the binary model of interval mapping, a single LOD peak with a maximum of 22.1 localized to LG14 in C2-50. LOD score and linkage group number is shown on the y- and x-axis, respectively. (**b**) The single LOD peak of 22.1 on LG14 is located at position 22.3 cM, which cosegregates with S14_4196799. The position of SNPs on LG14 is shown on the x-axis. LOD scores are displayed on the y-axis. The threshold was 2.89, as determined by 1000 permutations

The genetic maps constructed for *V. cinerea* C2-50 and Riesling contains 367 and 403 SNPs, respectively [28]. SNPs heterozygous in *V. cinerea* C2-50 and homozygous in Riesling were retained in the C2-50 SNP set, while the opposite set of SNPs were contained in the Riesling SNP set. To map *RDV2*, two genetic mapping strategies were performed. First, an interval mapping strategy was performed using R/qtl [29] with the C2-50 and Riesling SNP sets. In this analysis, the binary model of interval mapping was used to localize the *RDV2* locus, as chi-square analysis indicates that G1 and G4 grape phylloxera resistance is mediated by a single allele. To perform this analysis, resistant and susceptible F_1_ individuals were designated numerical values of 0 and 1, respectively. Results from the binary mapping showed that a single maximum LOD peak of 22.1 (*p*-value = 0.0) was identified on linkage group 14 (LG14) in C2-50 (Fig 1a). The maximum LOD peak cosegregated with S14_4196799 at position 22.3 cM (Fig 1b). The maximum LOD score value was above the threshold value of 2.89, as determined by 1000 permutations with alpha = 0.05. When the binary model of interval mapping was performed with the Riesling SNP set, LOD scores above the threshold value of 2.88 were not detected (Additional file 3).

**Fig. 2:**
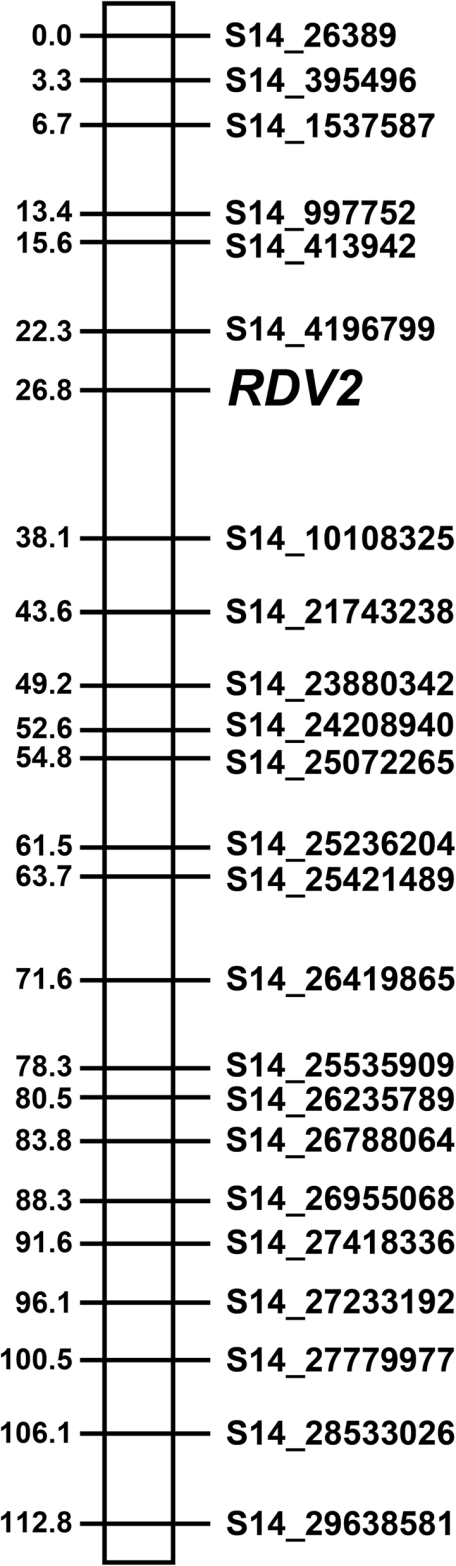
Genetic map of linkage group 14 with *RDV2* marker. Linkage mapping showing the location of *RDV2* on LG14 in C2-50. SNP ID and distance (cM) are shown on the right and left side of the LG14, respectively. All SNPs mapped to chromosome 14 in the PN40024 genome. The location of the SNPs in the PN40024 reference genome is indicated by the chromosome number (S14) followed by the position in bp

A linkage mapping approach was performed using R/OneMap [30], as a second strategy for localizing *RDV2*. In this approach, a pseudo-marker, called *RDV2*, was created in which resistant and susceptible F_1_ individuals were assigned either an *RDV2/rdv2* or *rdv2/rdv2* genotype, respectively. This was based on two observations: (1) chi-square analysis indicated that G1 grape phylloxera resistance is mediated by a single allele and (2) the binary model of interval mapping showed that *RDV2* mapped to a single locus with the C2-50 genetic map only. To map the *RDV2* locus, the *RDV2* marker was added to the C2-50 367 and Riesling 403 SNP sets and linkage analysis was performed. Results from the linkage mapping showed that the *RDV2* marker mapped to LG14 at 26.8 cM using the C2-50 SNP set (Fig 2). The *RDV2* marker was flanked by S14_4196799 and S14_10108325 at 22.3 and 38.1 cM, respectively (Fig 2). When the *RDV2* marker was included in the Riesling 403 SNP set, G1 grape phylloxera resistance was not mapped to any of the 19 linkage groups (data not shown). Taken together, the interval and linkage mapping studies showed that the *RDV2* locus localized to the vicinity of 22.3 to 26.8 cM on LG14 in C2-50.

### Validating SNPs at the *RDV2* locus

The TASSEL GBS pipeline [31] was used to identify SNPs from sequence tags aligned to the PN40024 reference genome in the 90 F_1_ *V. cinerea* C2-50 x Riesling individuals [28]. As the accuracy rate for predicting SNPs at the *MJR1* locus was 50% [28], we selected 42 SNPs spanning the *RDV2* locus and validated these markers using the Sequenom MassARRAY platform [32]. Results from this analysis showed that 22 out of the 42 SNPs genotyped were polymorphic and matched with those predicted by TASSEL (Table 3). For these 22 SNPs, the major and minor allele frequencies were nearly identical with those predicted by TASSEL (Table 3). The fact that these allele frequencies did not match completely was due to 11 genotyping errors in six F_1_ individuals (Table 4). The remaining 20 SNPs genotyped by Sequenom MassARRAY were non-polymorphic and did not match the genotypes predicted by TASSEL (Table 3). Taken together, results from Sequenom MassARRAY genotype validation indicate that the TASSEL GBS pipeline accurately predicted SNPs with a 52% success rate at the *RDV2* locus.

**Table 3:** Validation of 42 SNPs at the *RDV2* locus

		Sequenom MassARRAY			TASSEL		
SNP ID	Position	Genotype	MF	MAF	Genotype	MF	MAF
S14_2062712	2062712	GG/GT	0.72	0.28	GG/GT	0.72	0.28
S14_2846470	2846470	CC/TC	0.72	0.28	CC/TC	0.72	0.28
S14_3066185	3066185	AA/GA	0.72	0.28	AA/GA	0.72	0.28
S14_3222720	3222720	TT/TA	0.72	0.28	TT/TA	0.72	0.28
S14_3296164	3296164	CC/TC	0.72	0.28	CC/TC	0.72	0.28
S14_3596942	3596942	CC/CT	0.72	0.28	CC/CT	0.72	0.28
S14_4065142	4065142	TT/GT	0.72	0.28	TT/GT	0.72	0.28
S14_4196799	4196799	AA/AG	0.72	0.28	AA/AG	0.72	0.28
S14_4921219	4921219	CC/CT	0.74	0.26	CC/CT	0.74	0.26
S14_5274160	5274160	TT/CT	0.74	0.26	TT/CT	0.74	0.26
S14_5737727	5737727	AA/TA	0.74	0.26	AA/TA	0.74	0.26
S14_5771919	5771919	AA/CA	0.74	0.26	AA/CA	0.74	0.26
S14_5804788	5804788	AA/GA	0.74	0.26	AA/GA	0.74	0.26
S14_6008125	6008125	GG/CG	0.74	0.26	GG/CG	0.74	0.26
S14_6071298	6071298	GG/AG	0.74	0.26	GG/AG	0.74	0.26
S14_6071669	6071669	CC/CA	0.74	0.26	CC/CA	0.74	0.26
S14_6175917	6175917	TT/CT	0.74	0.26	TT/CT	0.74	0.26
S14_6596440	6596440	TT/TA	0.74	0.26	TT/TA	0.74	0.26
S14_7684469	7684469	AA/AG	0.74	0.26	AA/AG	0.75	0.25
S14_8894287	8894287	GG/GT	0.75	0.25	GG/GT	0.76	0.24
S14_9154944	9154944	TT/GT	0.76	0.24	TT/GT	0.76	0.24
S14_9705369	9705369	TT/CT	0.76	0.24	TT/CT	0.77	0.23
S14_3076105	3076105	TT	1	0	TT/TC	0.76	0.24
S14_3084705	3084705	GG	1	0	GG/GT	0.77	0.23
S14_3216815	3216815	CC	1	0	CC/CT	0.76	0.24
S14_3256262	3256262	GG	1	0	GG/GC	0.76	0.24
S14_3256274	3256274	AA	1	0	AA/AT	0.76	0.24
S14_3321418	3321418	CC	1	0	CC/CT	0.77	0.23
S14_3596980	3596980	TT	1	0	TT/TA	0.76	0.24
S14_3891954	3891954	CC	1	0	CC/CT	0.77	0.23
S14_4228151	4228151	TT	1	0	TT/TA	0.76	0.24
S14_6003431	6003431	CC	1	0	CC/CG	0.74	0.26
S14_7623091	7623091	GG	1	0	GG/GA	0.75	0.25
S14_7744603	7744603	AA	1	0	AA/AC	0.74	0.26
S14_7776601	7776601	GG	1	0	GG/GA	0.74	0.26
S14_7829543	7829543	AA	1	0	AA/AG	0.73	0.27
S14_7831615	7831615	CC	1	0	CC/CT	0.74	0.26
S14_7939992	7939992	CC	1	0	CC/CT	0.72	0.28
S14_8196757	8196757	AA	1	0	AA/AG	0.72	0.28
S14_8660835	8660835	GG	1	0	GG/GC	0.73	0.27
S14_9036371	9036371	GG	1	0	GG/GA	0.75	0.25
S14_9154939	9154939	GG	1	0	GG/GA	0.75	0.25

SNPs validated by Sequenom MassARRAY from 56 F_1_ individuals were compared with results from the TASSEL GBS data. Based on alignment with the PN40024 reference genome, SNPs from chromosome 14 were selected from position 2062712 to 9705369 for validation. The top section of the table contains 22 SNPs in which genotypes determined by Sequenom MassARRAY closely matched results produced by the TASSEL GBS pipeline. The bottom section lists SNPs genotyped by Sequenom MassARRAY, which did not match with results from the TASSEL GBS pipeline. *MF* Major Allele Frequency, *MAF* Minor Allele Frequency.

**Table 4:** Genotyping errors detected in F_1_ individuals after Sequenom MassARRAY genotyping

F_1_ Individual ID	SNP ID	Sequenom MassARRAY	TASSEL GBS Pipeline
k2b_16_06_6_6c	S14_9705369	CT	TT
k2b_16_08_6_6c	S14_9705369	CT	TT
k2b_16_11_5_6c	S14_9705369	CT	TT
k2b_16_13_2_6c	S14_2846470	CT	CC
	S14_5274160	CT	TT
	S14_6175917	CT	TT
	S14_9705369	CT	TT
k2b_16_13_7_6c	S14_7684469	AG	AA
k2b_16_15_1_6c	S14_4196799	AA	GA
	S14_7684469	AA	GA
	S14_9154944	TT	GT

### Genetic mapping with validated SNPs at the *RDV2* locus

To validate the previous genetic mapping results, a new genetic map for LG14 was created with the Sequenom MassARRAY genotyped SNPs from above. This was achieved by producing a 386 SNP set, which contained the 22 Sequenom MassARRAY genotyped SNPs. To create this SNP set, we first removed S14_997752 and S14_413942. Next, 21 of the accurately genotyped SNPs were added to the 367 SNP set (Note: S14_4196799 was part of the 367 SNP set; therefore, this marker did not need to be added to the final SNP set). Finally, the 11-genotyping errors identified in the six F_1_ individuals from the Sequenom MassARRAY validation results from above were corrected in the genotype file. Using R/OneMap, a new genetic map for LG14 containing the accurately genotyped SNPs was created (Additional file 4). Results showed that the final map size for LG14 was reduced from 112.8 to 101.5 cM (Additional file 4). In addition, six (S14_2846470, S14_3066185, S14_3222720, S14_3296164, S14_3596942 and S14_4065142) and nine (S14_5274160, S14_5737727, S14_5771919, S14_5804788, S14_6008125, S14_6071298, S14_6071669, S14_6175917, S14_6596440) SNPs cosegregated with S14_2062712 and S14_4921219 at 13.4 cM and 16.7, respectively (Additional file 4). In addition, S14_9154944 and S14_9705369 mapped to position 23.4 cM (Additional file 4). For interval and linkage mapping, 16 validated markers were removed from the 386 SNP set so that the final SNP set contained 370 markers including 6 validated markers, S14_2062712 (13.4 cM), S14_4196799 (14.5 cM), S14_4921219 (16.7 cM), S14_7684469 (21.2 cM), S14_8894287 (22.3 cM) and S14_9154944 (23.4 cM) (Additional file 5: Note this file also contains the *RDV2* marker).

**Fig. 3:**
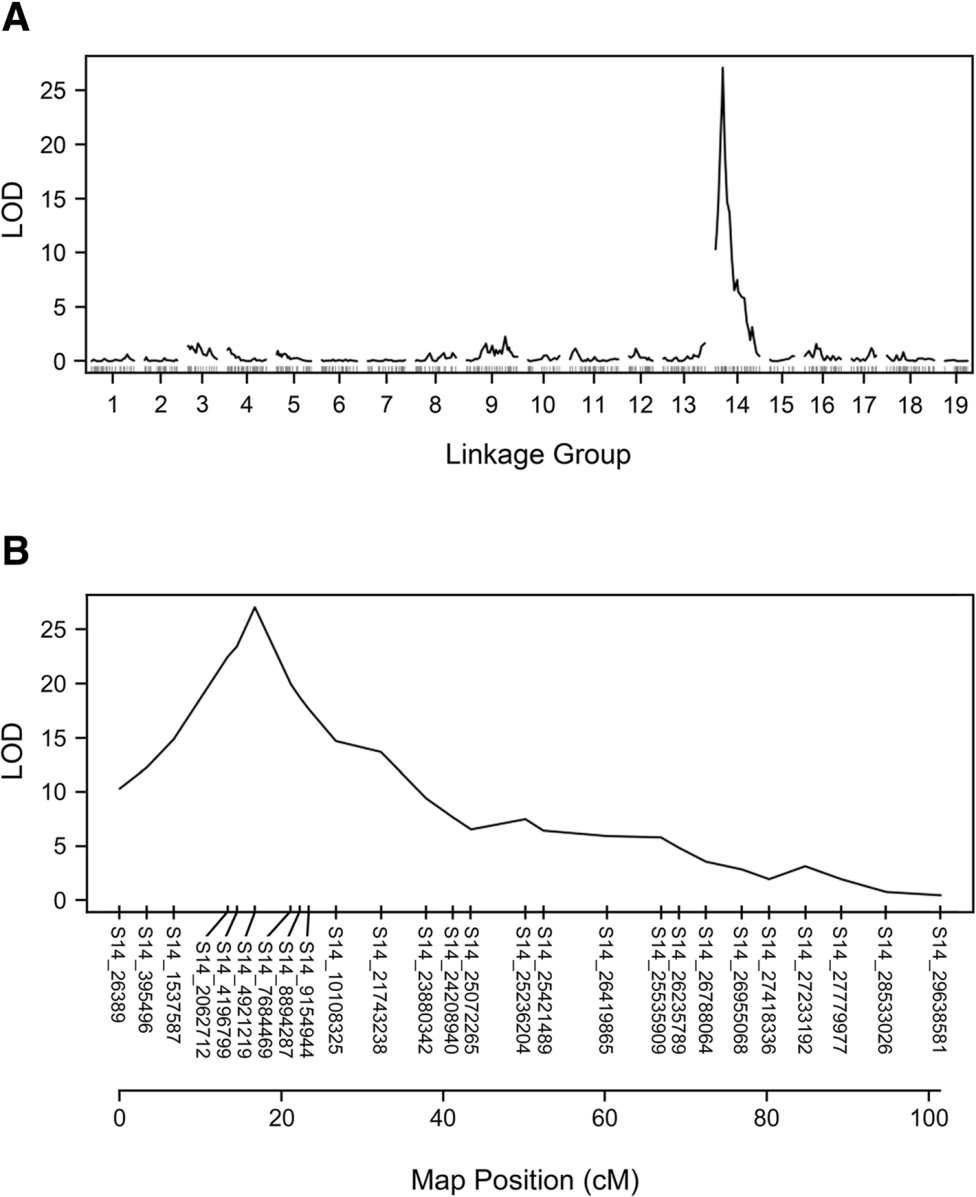
A binary model of interval mapping for *RDV2* using 6 validated SNPs. (**a**) A single maximum LOD peak of 27.1 localized to LG14 in C2-50. Linkage group number and LOD scores are shown on the x- and y-axis, respectively. (**b**) The LOD peak on LG14 at position 16.7 cM segregated with S14_4921219. The validated SNPs on LG14 were S14_2062712 (13.4 cM), S14_4196799 (14.5 cM), S14_4921219 (16.7 cM), S14_7684469 (21.2 cM), S14_8894287 (22.3 cM) and S14_9154944 (23.4). The position of SNPs on LG14 is shown on the x-axis. LOD scores are displayed on the y-axis. The threshold was 2.84, as determined by 1000 permutations

**Fig. 4:**
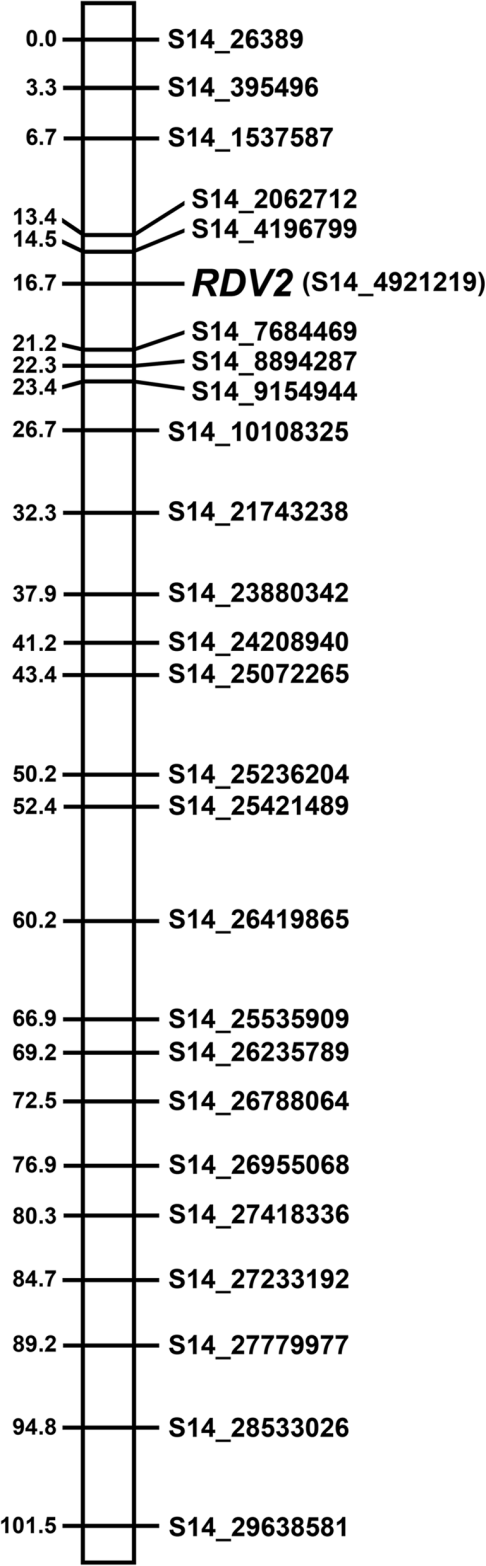
Linkage mapping of *RDV2* with the LG14 genetic map containing the 6 validated SNPs. The *RDV2* marker localized to LG14 in C2-50 at position 16.7 cM. S14_4921219 also localized to position 16.7 cM. SNP ID and distance (cM) are shown on the right and left side of the LG14, respectively. The eight validated SNPs are S14_2062712, S14_4196799, S14_4921219, S14_7684469, S14_8894287 and S14_9154944. All SNPs mapped to chromosome 14 in the PN40024 genome. The position of the SNP in the PN40024 reference genome is indicated by the by number following S14_

Using the binary model of interval mapping, a single maximum LOD peak of 27.1 (*p*-value = 0.0) was detected on LG14 with the C2-50 370 genetic map (Fig 3a). This peak was located at 16.7 cM and cosegregated with S14_4921219 (Fig 3b). The maximum LOD score of 27.1 was above the LOD threshold value of 2.84, as determined by 1000 permutations with alpha = 0.05. For linkage mapping, the *RDV2* marker was added to the C2-50 370 SNP set and results showed that this marker mapped to LG14 at 16.7 cM (Fig 4). The *RDV2* marker cosegregated with S14_4921219 and was flanked by S14_4196799 and S14_7684469 at positions 14.5 and 21.2 cM, respectively (Fig 4). Taken together, results from interval and linkage mapping support a model that *RDV2* is localized to LG14 at 16.7 cM.

## Discussion

A major component of effective vineyard management for grape phylloxera is due to the usage of phylloxera resistant rootstocks [3]. However, the majority of commercial rootstocks available for production are partially resistant (or tolerant) to grape phylloxera feeding and reproduction. Therefore, identifying and mapping new sources of grape phylloxera resistant loci and validating linked molecular markers provide valuable tools for the introgression of multiple levels of phylloxera resistance into new rootstocks via marker-assisted selection. Experimental studies directed at understanding the genetic control of grape phylloxera resistance indicate that resistance is controlled by multiple loci in *V. berlandieri*, *V. cinerea*, *V. rupestris* and *M. rotundifolia* [10, 22–24]. In this study, *V. cinerea* C2-50, a *V. aestivalis* accession, DRX55 and MS27-31 were identified as sources of G1 phylloxera resistance. Genetic analysis of the F_1_ *V. cinerea* C2-50 x Riesling population indicated that G1 and G4 grape phylloxera resistance is controlled by a single allele in which C2-50 is heterozygous dominant for *RDV2* (*RDV2/rdv2*). Furthermore, validation of SNPs at the *RDV2* locus by Sequenom MassARRAY followed by linkage and interval mapping support a model that this grape phylloxera resistant locus is located on chromosome 14 at position 16.7 cM. The validated SNPs that cosegregate and flank the *RDV2* locus are essential genetic determinants that can be used for marker-assisted breeding of new rootstocks with phylloxera resistance.

As *RDV1*, which is derived from *V. cinerea* Arnold, is located on chromosome 13 in the Börner rootstock [25], the mapping of *RDV2* to chromosome 14 in *V. cinerea* C2-50 indicates that the *V. cinerea* contains at least two grape phylloxera resistant loci. In MN1264, a leaf specific-grape phylloxera resistance locus maps to chromosome 14 between 10-30 cM [26], which overlaps with the location of *RDV2* in *V. cinerea* C2-50. In contrast, resistance to the root form of phylloxera mapped to chromosome 5 and 10 in MN1264 and MN1246, respectively [26]. As the pedigree of MN1264 does not contain *V. cinerea* [26], it appears that the leaf specific-grape phylloxera resistant locus in this complex hybrid may be distinct from *RDV2* in C2-50. Taken together, four root grape phylloxera resistance loci have been identified in grapevine, all of which map to different chromosome. If the mechanism of grape phylloxera resistance is distinct for each of these four loci, then durable resistance could be achieved by stacking at least two traits into a single rootstock cultivar. Moreover, the identification of markers linked to the root knot nematode resistant locus, *MELOIDOGYNE JAVANICA ‘PT 1103P’ RESISTANT 1* (*MJR1*) [28] together with SNPs linked to *RDV2* show that *V. cinerea* C2-50 is a valuable breeding line for marker-assisted selection of new rootstocks with both grape phylloxera and root knot nematode resistance.

Grape phylloxera has been categorized into seven biotypes (A-G) based on grape phylloxera-host plant interactions [16]. These interactions involve host plant responses, including nodosity, tuberosity and/or pseudotuberosity formation, as well as immune defense responses, all of which are dependent upon the genotype of the plant and the biotype group. The geographical distribution and size of the biotype population in viticulture regions appears to be linked in part to the genotype of the root (rootstock or own rooted *V. vinifera*) used in viticulture production. For example, ‘biotype C’ is the predominant group in Europe [16], which is likely due to the widespread usage of rootstocks with *V. riparia* parentage [15, 20]. In Australia, the major genetic strains, G1 and G4, belong to ‘biotype A’, which is likely due to the relatively low to moderate rate of rootstock adoption and the widespread use of own rooted *V. vinifera* vines [3, 16]. However, biotypes less adapted to feeding and reproducing on the predominant root systems used in viticulture production may still exist in low numbers in the soil and have been observed on both *V. vinifera* and rootstock roots in Australian Phylloxera Infested Zones (PIZs) [33]. As a result, the emergence of a less endemic biotypes is a major problem when management practices utilize rootstocks with partial resistance and/or a limited spectrum of resistance. *RDV1* was identified by screening F_1_ *V. vinifera* V3125 x Börner individuals for resistance to grape phylloxera sourced from the leaves of *V. berlandieri* x *V. riparia* rootstocks, SO4 and 125 AA [25]. Therefore, it is likely that the grape phylloxera used in this screening assay were of the ‘biotype C’ group, genetic strains adapted to feeding on rootstocks with *V. riparia* parentage [16]. In contrast, the G1 and G4 genetic strains, which fall into the ‘biotype A’ group [16], were used to identify *RDV2* locus. As complex hybrids were utilized for mapping phylloxera resistance loci in MN1264 and MN1246 [26], it is difficult to predict the biotype(s) used in their screening assay. Using the *in planta*-potted bioassay, Börner was shown to be resistant to G1 and G4, as well as four other Australian genetic strains [3, 34]; however, it is not clear if this broad spectrum of resistance to the six genetic strains is mediated by *RDV1*. In addition, preliminary results indicate that *V. cinerea* C2-50 provides resistance to the G30 genetic strain (Smith HM and Powell KS, unpublished), but whether this is mediated by *RDV2* is unclear. Therefore, future work is aimed at determining the spectrum of grape phylloxera biotype resistance for *RDV1* and *RDV2*.

## Conclusion

In the present study, we have identified grapevine-breeding material, including *V. cinerea* C2-50 and a *V. aestivalis* accession, as well as the DRX55 and MS27-31 *M. rotundifolia* hybrids, that have complete resistance to G1 grape phylloxera. Using the C2-50 genetic map derived from the F_1_ *V. cinerea* C2-50 x Riesling mapping population, a single locus for G1 phylloxera resistance, *RDV2*, was identified on chromosome 14. After validating SNPs, *RDV2* was localized to position 16.7 cM on chromosome 14. Based on the previous identification of *RDV1* [25] and the mapping of *RDV2*, *V. cinerea* may contain at least two phylloxera resistant loci. Validated SNPs at the *RDV2* locus will serve as valuable tools for the marker-assisted selection of new rootstocks aimed at improving vineyard performance. Furthermore, if the mechanism of *RDV1* and *RDV2* mediated grape phylloxera resistance is different markers linked to these loci will be useful for combining these traits into a single rootstock for durable resistance.

## Methods

### Phylloxera Stock Cultures

Biotype A grape phylloxera strains, G1 and G4, were selected for screening, as these two strains are highly aggressive and genetically similar to each other compared to other phylloxera strains identified in Australia [35, 36]. Moreover, G1 is the most geographically widespread phylloxera strain in Australia. G1 and G4 grape phylloxera were single sourced from the roots of ungrafted *V. vinifera* vines in commercial vineyards located in the PIZs of Maroondah and North East Victoria respectively. Prior to the screening trial, G1 and G4 were maintained and multiplied on *V. vinifera* cv. Chardonnay excised root pieces under controlled conditions in growth cabinets (25±2°C, 12 hours light) using a recommended excised root bioassay procedure [16, 37]. Grape phylloxera strains were sub-sampled and genotyped at Agribio, Bundoora, before and after the trial, using six microsatellite markers [36]. All phylloxera stock strains were maintained under strict quarantine conditions in a laboratory at Agriculture Victoria, Rutherglen located in the North East PIZ.

### Planting Material and growth conditions

The grape phylloxera screening trial utilized six-week old potted plants propagated from dormant cuttings by Yalumba Nursery (Nuriootpa, South Australia). All plants were potted in 80% sterile potting mix plus 20% Perlite for adequate soil aeration and watered via drip irrigation for at least six weeks, in a controlled-temperature glasshouse, to enable good root development prior to phylloxera inoculation. Each genotype was screened in triplicate with G1 grape phylloxera. Fifty-eight out of the 90 F_1_ *V. cinerea* C2-50 x Riesling individuals were also screened with G4 grape phylloxera. At trial commencement, all vines were fertilized with 3.5 g Osmocote^TM^ and 500 ml Thrive (8 g of Thrive^TM^ mixed with 4.5 L water) per potted vine. Each vine was drip irrigated for two minutes every two days. To prevent the vines from going into dormancy, artificial growth lights were automatically turned on each day from 6am to 8pm and 1am to 2am during the trial. During the trials, Gemini Tinytag Ultra^TM^ dataloggers (Hastings Data Loggers, Port Macquarie, New South Wales) monitored the temperature and relative humidity in the glasshouse. The temperature settings were minimum 20^0^C and maximum 24^0^C from 8am to 9pm, min 20°C and maximum 25^0^C from 9pm-8am.

### *In planta*-potted phylloxera screening assay

For phylloxera infestation, each vine was removed from the pot and the roots were cleaned of potting mix. Next, a single lignified root piece approximately 2.75 cm in diameter was selected and a moistened filter paper strip containing twenty phylloxera eggs was carefully wrapped around the exposed root. This method of enclosing the phylloxera around a lignified root piece is similar to the root enclosure *in planta* bioassay method described by Korosi *et al.* (2007). This is one of the methods recommended as a standardised potted plant bioassay protocol to allow comparative phenological observations induced by different phylloxera biotypes [16]. The infested vine was placed in a sterile 8 cm diameter pot with the addition of fresh sterile soil mixture where required (80% potting mix, 20% perlite). Tanglefoot® insect barrier was placed around the base of the stem and the rim of each pot to avoid cross contamination of phylloxera between vines. Eight weeks after infestation, resistance and susceptibility to G1 and G4 grape phylloxera was determined by first clipping the stem near the soil surface and discarding the shoot. Next, roots were carefully removed from the pots and rinsed with approximately 100ml of water. The 100ml root washing was passed through a 53μm sieve and the number of insects was scored in the filtrated water sample. Next, the number of insects and nodosities were counted on the roots by microscopic examination. The final number of insects scored for each replicate was determined by adding the number of insects on the roots with number in the root washing. The average number of nodosities and insects was determined by screening three propagated vines per genotype. Grapevine genotypes with an average score of nodosities and insects equal to zero were classified as resistant, while plants with >0 nodosities and insects were classified as susceptible. All phylloxera infested grapevine material was maintained under strict quarantine conditions in a secure glasshouse facility at Agriculture Victoria, Rutherglen located in the North East PIZ.

### Trial design

A randomized complete plot design was established to examine the interaction between G1 and G4 grape phylloxera and individual vine genotypes used in this study. Three replicate vines were used per treatment in a block design to account for any variation in the environmental conditions.

### Genetic mapping of the grape phylloxera resistance

A genotyping-by-sequence approach [38] followed by the TASSEL SNP calling pipeline [31] was used to identify the SNPs in the F_1_ *V. cinerea* C2-50 x Riesling individuals [28]. Genetic maps were constructed using R/OneMap [30] with 367 and 403 SNPs for C2-50 and Riesling, respectively [28]. Note: the C2-50 genetic map was constructed from SNPs that were heterozygous in C2-50 and homozygous in Riesling while the opposite set of SNPs were used to generate the Riesling genetic map. The phylloxera resistant locus was identified by linkage and interval mapping. Using the Kosambi function for linkage mapping in R/OneMap [30], SNPs were ordered with a LOD of 6.0 and a recombination frequency of 0.25. Using R/qtl [29], the binary model of interval mapping was performed by first converting the phenotype data to a numerical binary trait. Next, a one-dimensional genome scan using the *scanone* function was performed with the argument *model = binary*. The LOD threshold value was estimated with 1000 permutations with *alpha = 0.05*. The name of each SNP provides chromosome and position information based on the PN40024 reference genome [39, 40]. For example, the SNP called S14_4196799 is located on chromosome 14 (S14) at position 4196799 bp.

### Sequenom MassARRAY validation of SNPs at the phylloxera resistance locus

The C2-50 367 SNP set was curated from a larger SNP set of 3974 markers produced by genotyping-by-sequencing the F_1_ *V. cinerea* C2-50 x Riesling individuals [28]. Forty-two SNPs from position 2062712 to 9705369 on chromosome 14 in the PN40024 genome were selected for SNP validation. DNA was isolated from 56 out of the 90 F_1_ C2-50 x Riesling individuals using the NucleoSpin® Plant DNA extraction kit (http://www.mn-net.com). The genomic DNA samples were sent to the Australian Genome Research Facility for SNP genotyping. This service utilized the SNP genotyping Sequenom MassARRAY iPLEX platform (Sequenom, San Diego, CA, USA) [32]. A comparison of the genotype data from Sequenom MassARRAY and the TASSEL SNP calling pipeline was performed to validate SNPs.

## Additional files

Additional file 1: Evaluation of G1 grape phylloxera resistance**.** The average number of G1 nodosities and insects were graphically represented for the 90 F_1_ individuals. (PDF 287 kb)

Additional file 2: Evaluation of G4 grape phylloxera resistance. For G4 phylloxera resistance, a graphic representation of the average number of nodosities and insects for 58 F_1_ individuals were shown in this file. (PDF 306 kb)

Additional file 3: Interval mapping of grape phylloxera resistance using the Riesling genetic map. The binary model of interval mapping for grape phylloxera resistance using the Riesling SNP set. This graph shows the LOD scores across the 19 linkage groups in Riesling. (PDF 86 kb)

Additional file 4: **Table S1.** Genetic map of LG14 using the 22 Sequenom MassARRAY validated SNPs. (PDF 44 kb)

Additional file 5: *RDV2* mapping file. The R/OneMap file used to determine the location of *RDV2* in Fig. 4. This mapping file contains the 6 validated SNPs spanning the *RDV2* locus, as well as the *RDV2* marker. In addition, the genotyping errors displayed in Table 4, were corrected in this file. (TXT 88 kb)

## Abbreviations

GBS: Genotyping-by-sequence
RDV1: Resistance daktulosphaira vitifoliae 1
RDV2: Resistance to daktulosphaira vitifoliae 1
SNP: Single nucleotide polymorphism
