# Supplementary material for: Genetic identification of SNP markers linked to a new grape phylloxera resistant locus in Vitis cinerea for marker-assisted selection
Source: BMC Plant Biol. 2018 Dec 18;18:360. doi: 10.1186/s12870-018-1590-0 (PMC6299647; doi:10.1186/s12870-018-1590-0)
Supplement: Supplementary file 1 — Evaluation of G1 grape phylloxera resistance. The average number of G1 nodosities and insects were graphically represented for the 90 F1 individuals. (PDF 287 kb) [file 12870_2018_1590_MOESM1_ESM.pdf]

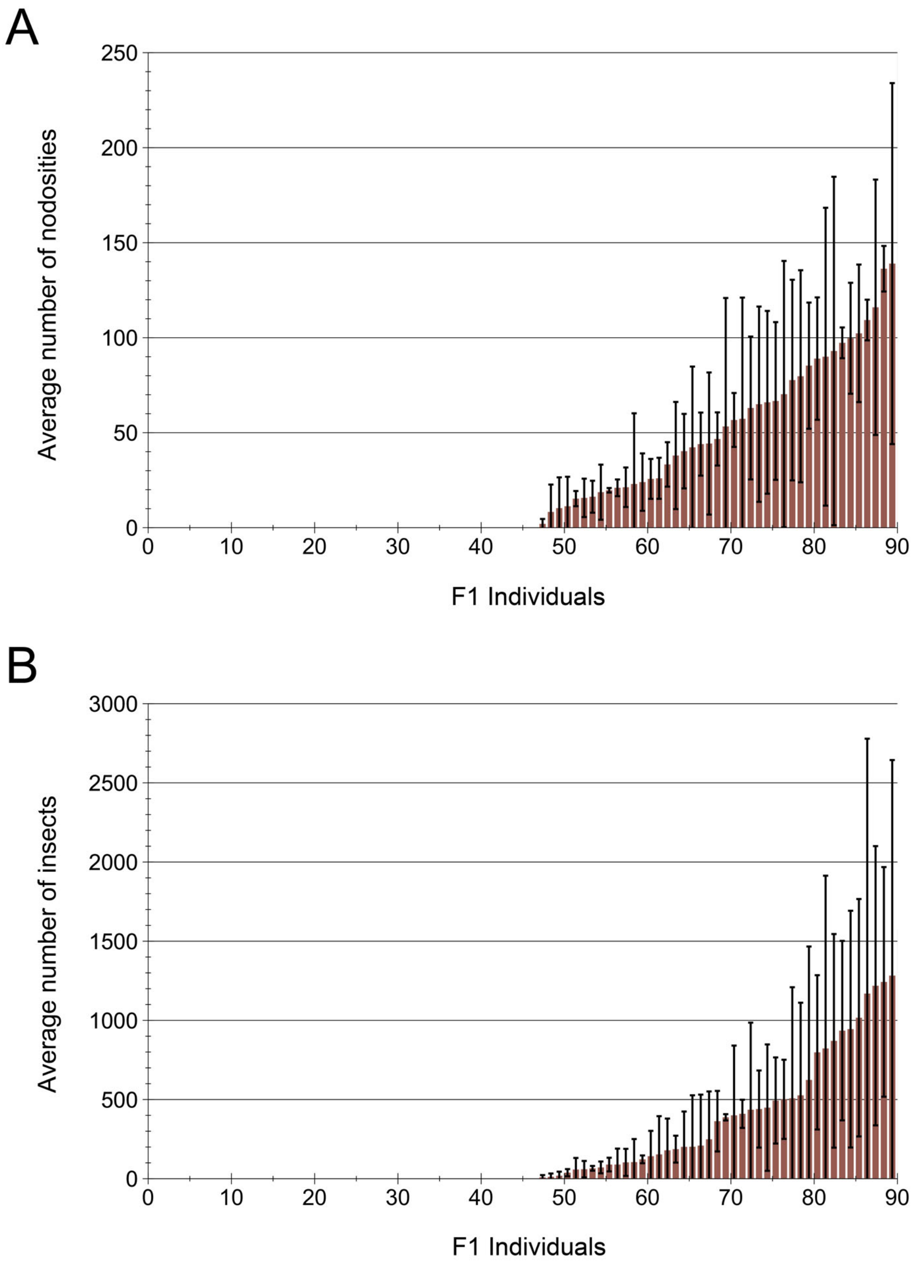


**Additional file 1: Evaluation of G1 grape phylloxera resistance**
After infestation with G1 grape phylloxera, (A) the average number of nodosities and (B) insects were determined by screening three propagated vines per $F_1$ individual.
