# Supplementary material for: Genetic identification of SNP markers linked to a new grape phylloxera resistant locus in Vitis cinerea for marker-assisted selection
Source: BMC Plant Biol. 2018 Dec 18;18:360. doi: 10.1186/s12870-018-1590-0 (PMC6299647; doi:10.1186/s12870-018-1590-0)
Supplement: Supplementary file 2 — Evaluation of G4 grape phylloxera resistance. For G4 phylloxera resistance, a graphic representation of the average number of nodosities and insects for 58 F1 individuals were shown in this file. (PDF 306 kb) [file 12870_2018_1590_MOESM2_ESM.pdf]

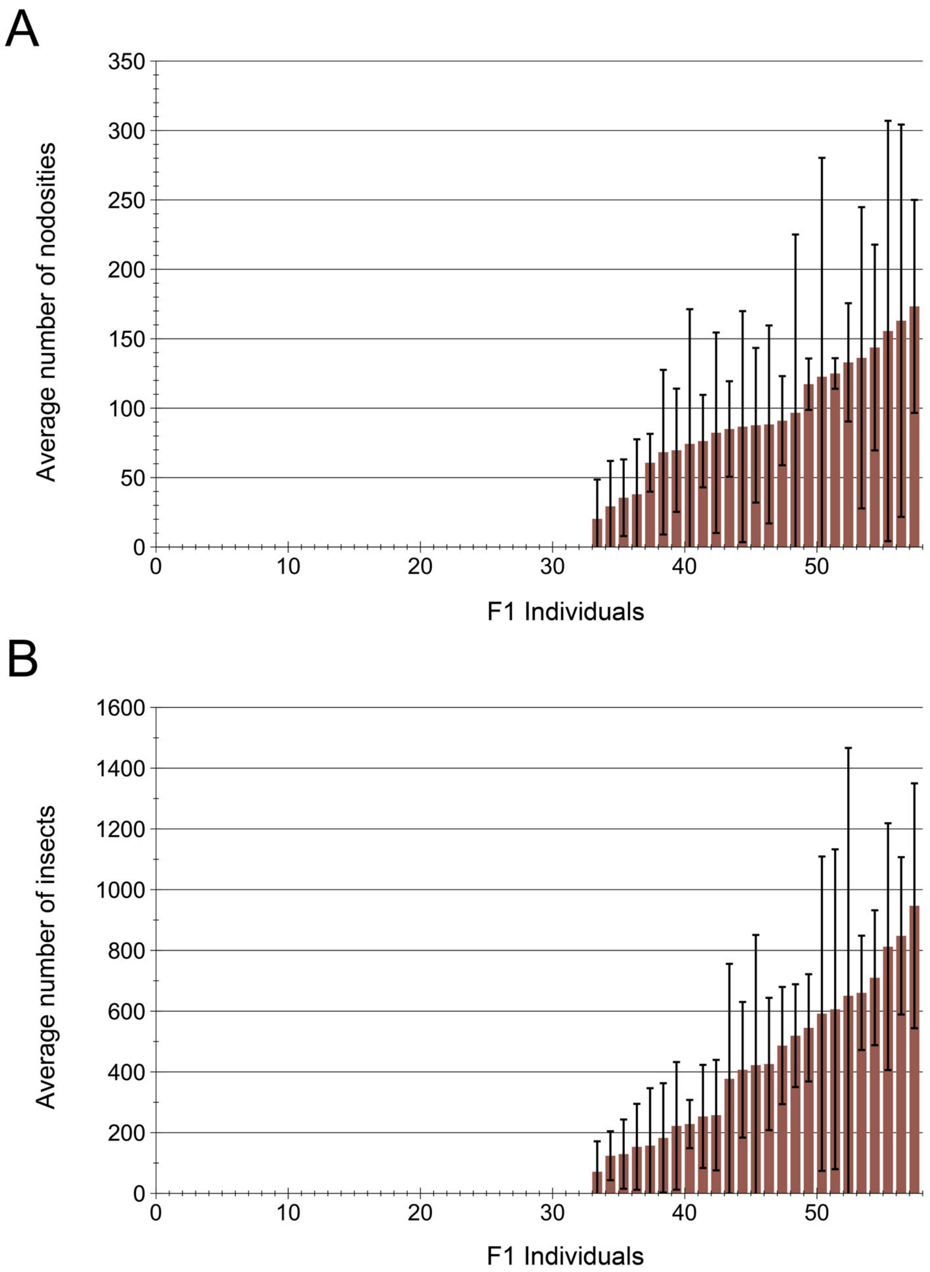

Additional file 2: Evaluation of G4 grape phylloxera resistance in 58 F_1 individuals

(A) The average number of nodosities and (B) insects were calculated for three propagated vines per F_1 individual after infestation with G4 grape phylloxera.
