# Supplementary material for: Genetic identification of SNP markers linked to a new grape phylloxera resistant locus in Vitis cinerea for marker-assisted selection
Source: BMC Plant Biol. 2018 Dec 18;18:360. doi: 10.1186/s12870-018-1590-0 (PMC6299647; doi:10.1186/s12870-018-1590-0)
Supplement: Supplementary file 3 — Interval mapping of grape phylloxera resistance using the Riesling genetic map. The binary model of interval mapping for grape phylloxera resistance using the Riesling SNP set. This graph shows the LOD scores across the 19 linkage groups in Riesling. (PDF 86 kb) [file 12870_2018_1590_MOESM3_ESM.pdf]

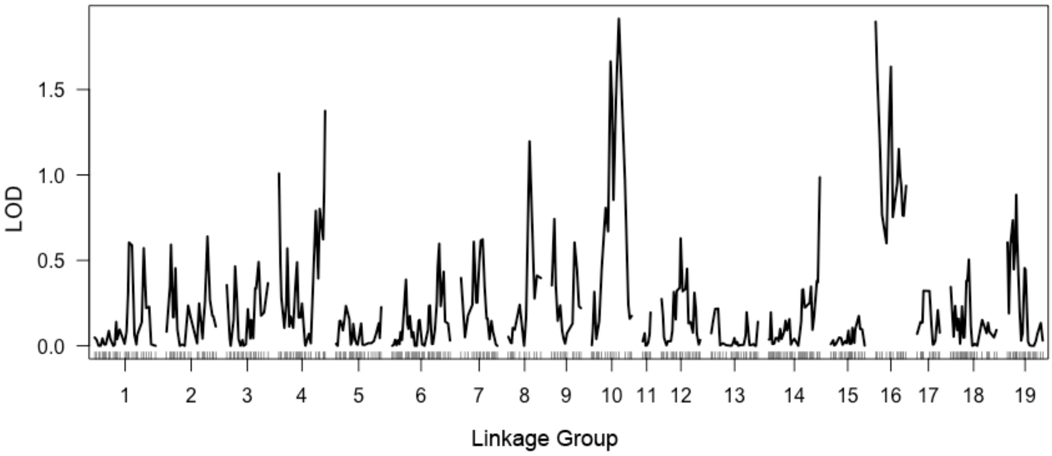

Additional file 3: Interval mapping of grape phylloxera resistance using the Riesling genetic map

Using the binary model, no LOD peaks above the threshold for phylloxera resistance were detected with the Riesling genetic map. The LOD threshold was 2.88, as determined by 1000 permutations. LOD score and linkage group number is shown on the y- and x-axis, respectively.
