# Supplementary material for: Genetic identification of SNP markers linked to a new grape phylloxera resistant locus in Vitis cinerea for marker-assisted selection
Source: BMC Plant Biol. 2018 Dec 18;18:360. doi: 10.1186/s12870-018-1590-0 (PMC6299647; doi:10.1186/s12870-018-1590-0)
Supplement: Supplementary file 4 — Table S1. Genetic map of LG14 using the 22 Sequenom MassARRAY validated SNPs. (PDF 44 kb) [file 12870_2018_1590_MOESM4_ESM.pdf]

Table S1: Genetic map of LG14 with the 22 Sequenom MassARRAY validated SNPs

SNP_ID	cM
S14_26389	0
S14_395496	3.3
S14_1537587	6.7
S14_2062712	13.4
S14_2846470	13.4
S14_3066185	13.4
S14_3222720	13.4
S14_3296164	13.4
S14_3596942	13.4
S14_4065142	13.4
S14_4196799	14.5
S14_4921219	16.7
S14_5274160	16.7
S14_5737727	16.7
S14_5771919	16.7
S14_5804788	16.7
S14_6008125	16.7
S14_6071298	16.7
S14_6071669	16.7
S14_6175917	16.7
S14_6596440	16.7
S14_7684469	21.2
S14_8894287	22.3
S14_9154944	23.4
S14_9705369	23.4
S14_10108325	26.7
S14_21743238	32.3
S14_23880342	37.9
S14_24208940	41.2
S14_25072265	43.4
S14_25236204	50.2
S14_25421489	52.4
S14_26419865	60.2
S14_25535909	66.9
S14_26235789	69.2
S14_26788064	72.5
S14_26955068	76.9
S14_27418336	80.3
S14_27233192	84.7
S14_27779977	89.2
S14_28533026	94.8
S14_29638581	101.5

The 22 validated SNPs in the LG14 map included markers from S14_2062712 to S14_9705369.
